# Complement Component 5 (C5) Deficiency Improves Cognitive Outcome After Traumatic Brain Injury and Enhances Treatment Effects of Complement Inhibitors C1-Inh and CR2-Crry in a Mouse Model

**DOI:** 10.1089/neur.2023.0024

**Published:** 2023-10-11

**Authors:** Min Chen, Stephen R. Edwards, Dhiraj Maskey, Trent M. Woodruff, Stephen Tomlinson, David Reutens

**Affiliations:** ^1^ Centre for Advanced Imaging, The University of Queensland, St Lucia, Queensland, Australia; ^2^ School of Biomedical Sciences, Faculty of Medicine, The University of Queensland, St Lucia, Queensland, Australia; ^3^ Queensland Brain Institute, The University of Queensland, St Lucia, Queensland, Australia; ^4^ College of Medicine, Medical University of South Carolina, Charleston, South Carolina, USA; ^5^ Australian Research Council Training Centre for Innovation in Biomedical Imaging Technology, The University of Queensland, St Lucia, Queensland, Australia

**Keywords:** C1-Inh, C5 deficiency, complement system, CR2-Crry, PMX205, traumatic brain injury

## Abstract

A potent effector of innate immunity, the complement system contributes significantly to the pathophysiology of traumatic brain injury (TBI). This study investigated the role of the complement cascade in neurobehavioral outcomes and neuropathology after TBI. Agents acting at different levels of the complement system, including 1) C1 esterase inhibitor (C1-Inh), 2) CR2-Crry, an inhibitor of all pathways acting at C3, and 3) the selective C5aR1 antagonist, PMX205, were administered at 1 h post-TBI. Their effects were evaluated on motor function using the rotarod apparatus, cognitive function using the active place avoidance (APA) task, and brain lesion size at a chronic stage after controlled cortical impact injury in C5-sufficient (C5^+/+^) and C5-deficient (C5^–/–^) CD1 mice. In post-TBI C5^+/+^ mice, rotarod performance was improved by CR2-Crry, APA performance was improved by CR2-Crry and PMX205, and brain lesion size was reduced by PMX205. After TBI, C5^–/–^ mice performed better in the APA task compared with C5^+/+^ mice. C5 deficiency enhanced the effect of C1-Inh on motor function and brain damage and the effect of CR2-Crry on brain damage after TBI. Our findings support critical roles for C3 in motor deficits, the C3/C5/C5aR1 axis in cognitive deficits, and C5aR1 signaling in brain damage after TBI. Findings suggest the combination of C5 inhibition with C1-Inh and CR2-Crry as potential therapeutic strategies in TBI.

## Introduction

Severe traumatic brain injury (TBI) causes significant chronic neurological, cognitive, and behavioral disability, resulting in an immense socioeconomic burden. Effective treatments are required to combat the chronic effects of TBI. Inflammatory mechanisms have been identified as an important treatment target because of their crucial role in secondary damage and the spread of pathology into regions surrounding the primary injury site.^1^ Robust and persistent activation of the complement system, a critical component of the innate immune system, contributes to neuroinflammation and the deleterious sequelae of TBI.^2–5^ Targeting the complement system is a potential therapeutic target for developing future treatments for TBI.

The complement system comprises >40 proteins involved in a series of enzymatic cleavages and membrane binding events. It plays a critical role in clearing pathogens, dying cells, and misfolded proteins and can be activated by the classical, lectin, or alternative pathways, resulting in activation of common major effectors. Complement components 3 and 5 (C3 and C5) lie at the center of the complement system.^6^ C3 is cleaved to form C3a, which promotes chemotaxis and activation of microglia through the receptor C3aR, and C3b which mediate opsonization. Cleavage of C5 mediates proinflammatory effects by: 1) generation of the C5a anaphylatoxin that, primarily through its G-protein-coupled receptor C5aR1, initiates inflammatory responses including recruitment and activation of inflammatory cells, blood–brain barrier disruption, and cytokine release; 2) the terminal pathway, which culminates in the formation of the cytolytic membrane attack complex (MAC).^7^

Dysregulation at different levels of the complement cascade may have different effects on neuropathological and -behavioral outcomes post-TBI. Hence, we hypothesized that the administration, after TBI, of agents targeting different levels of the complement cascade have different effects on motor and cognitive function and brain damage.

To test this hypothesis, we used three inhibitors that act at different levels of the complement system (depicted in Fig. 2B):

1. C1 esterase inhibitor (C1-Inh). C1-Inh is a member of the serpin family of protease inhibitors, which inhibits the complement system, contact (kinin) system, and fibrinolytic/coagulation system.^8^ Human-derived C1-Inh, a U.S. Food and Drug Administration (FDA)-approved drug, is active in mice; hence, it can be tested in murine models.^9^
2. Complement receptor 2-complement receptor 1–related gene/protein (CR2-Crry). Crry is a membrane complement regulator that inhibits C3 convertase. A CR2-targeting moiety targets Crry to the site of complement activation and C3d deposition,^10,11^ allowing recombinant CR2-Crry to act as a C3 inhibitor at the injury site.
3. PMX205. This is a small, orally active and brain permeable cyclic peptide (HC-[OPdChaWR] that acts as a non-competitive antagonist of the complement C5a (C5a) receptor (complement C5a receptor 1; C5aR1).^12–14^ The inclusion of a C5aR1 antagonist allowed us to determine whether any observed benefit of C5aR1 blockade in TBI is mediated through the attenuation of C5a-C5aR1 signaling.

Up to 39% of murine strains, including C57BL/10Sn, DBA/2J, A/HeJ, AKR/J, NZB/BINJ, SWR/J, and B10.D2/oSnJ, have been reported to be C5 deficient and to lack detectable blood levels of C5.^15^ In C5-deficient mouse strains, the 2-base-pair “TA” deletion at positions 661 and 662 of the C5 messenger RNA (mRNA) coding frame results in failure to secrete C5 protein, although it is biosynthesized.^15^ C5-deficient mouse strains represent valuable tools for investigating the role of C5 in TBI.^16^ We hypothesized that the absence of C5 affects functional outcomes after TBI and influences the therapeutic effects of complement inhibitors by comparing them in C5^+/+^ and C5^–/–^ CD1 mice, an outbred strain that is commonly used for testing the efficacy and safety of new drugs in TBI and post-traumatic epilepsy models.

## Methods

### Experimental design

**FIG. 1.**
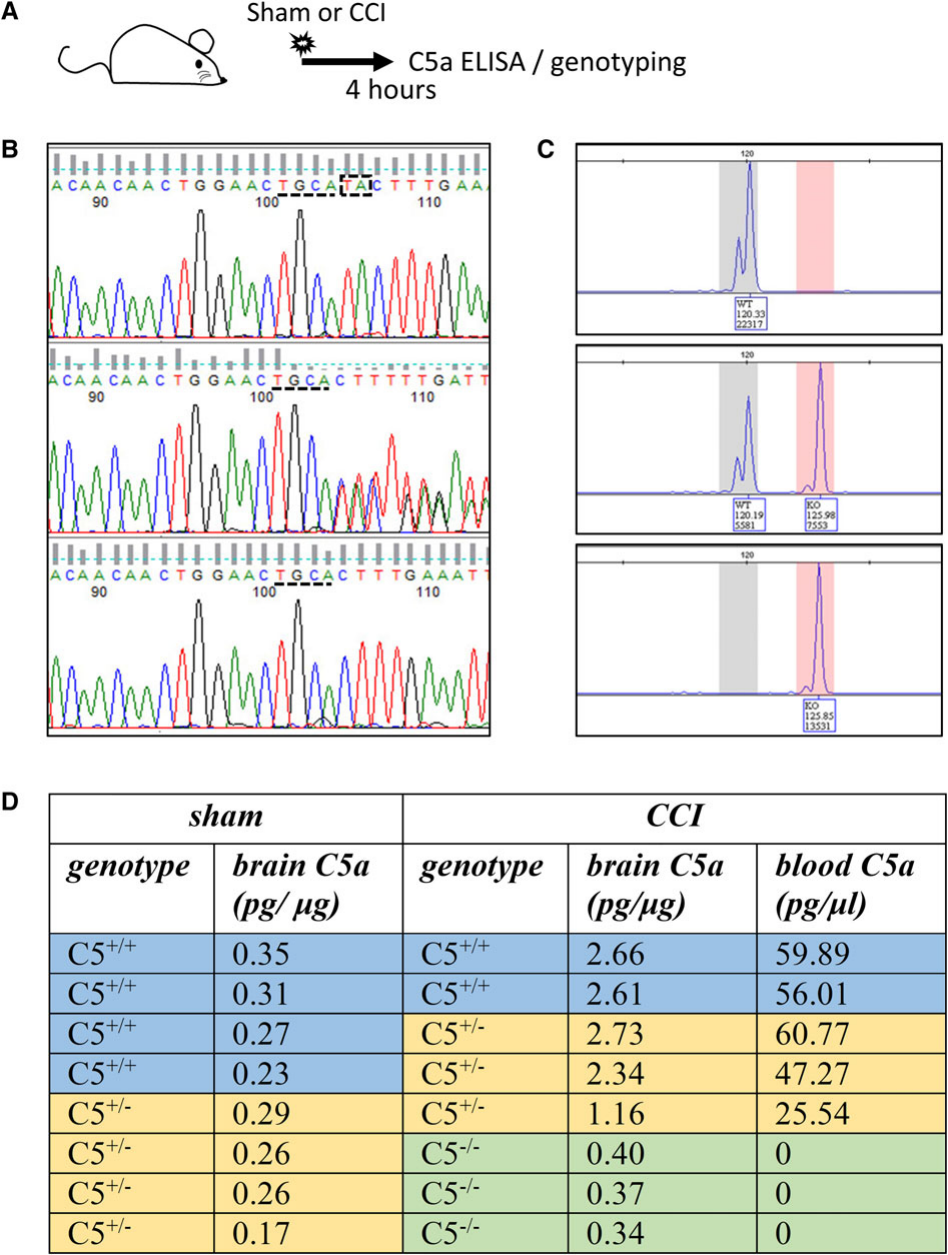
C5 deficiency in outbred CD1 mice. (**A**) Schematic representation of the timeline for the experimental procedures. Screen shots of representative data for sequencing (**B**) and MS-PCR (**C**) from C5^+/+^ (top), C5^+/–^ (middle), and C5^–/–^ (bottom) outbred CD1 mice. In the sequencing screenshots, the TA base-pair that followed TGCA after sequence number 100 was deleted in the mutational C5 allele. The MS-PCR screenshots showed that PCR fragments with two different sizes were present in C5^+/+^, C5^+/–^, and C5^–/–^ mice. (**D**) Brain C5a levels in sham and CCI-injured C5^+/+^, C5^+/–^, and C5^–/–^ mice 4 h after injury. C1-Inh, C1 esterase inhibitor; CCI, controlled cortical impact; ELISA, enzyme-linked immunosorbent assay; MS-PCR, mutagenically separated polymerase chain reaction.

**FIG. 2.**
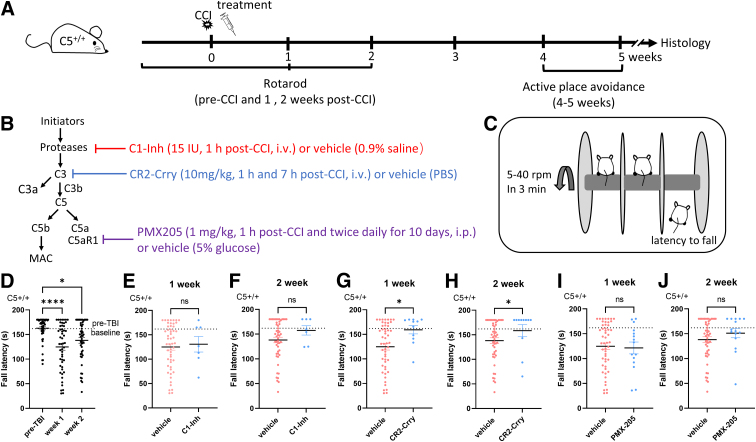
Effects of complement inhibitors on rotarod performance in C5^+/+^ mice, at 1 and 2 weeks after CCI injury. (**A**) Schematic representation of the timeline for the experimental procedures and drug administration. (**B**) Summary diagram of the complement pathways and site of action of the inhibitors in C5^+/+^ animals. (**C**) Setup of the rotarod test. (**D**) Fall latency of the rotarod test was significantly lower at 1 and 2 weeks after CCI compared to pre-treatment baseline in C5^+/+^ mice (*n* = 52; Kruskal-Wallis' test, ****p < 0.0001; Dunn's multiple comparisons test, *p < 0.05, ****p < 0.0001). (**E,F**) Compared with vehicle, C1-Inh treatment did not significantly increase fall latency over the 2-week period after CCI injury in C5^+/+^ mice (C1-Inh, *n* = 7; vehicle, *n* = 52; Mann-Whitney U test, *p* = 0.85 at 1 week post-CCI and *p* = 0.25 at 2 weeks post-CCI). (**G,H**) Compared with vehicle, CR2-Crry treatment significantly increased fall latency at 1 and 2 weeks after CCI injury in C5^+/+^ mice (CR2-Crry, *n* = 11; vehicle, *n* = 52; Mann-Whitney U test, *p < 0.05). (**I,J**) Compared with vehicle, PMX205 treatment did not significantly increase fall latency over the 2-week period after CCI injury in C5^+/+^ mice (PMX205, *n* = 15; vehicle, *n* = 52; Mann-Whitney U test, *p* = 0.74 at 1 week post-CCI and *p* = 0.24 at 2 weeks post-CCI). C1-Inh, C1 esterase inhibitor; CCI, controlled cortical impact; CR2-Crry, complement receptor 2-complement receptor 1–related gene/protein.

**FIG. 3.**
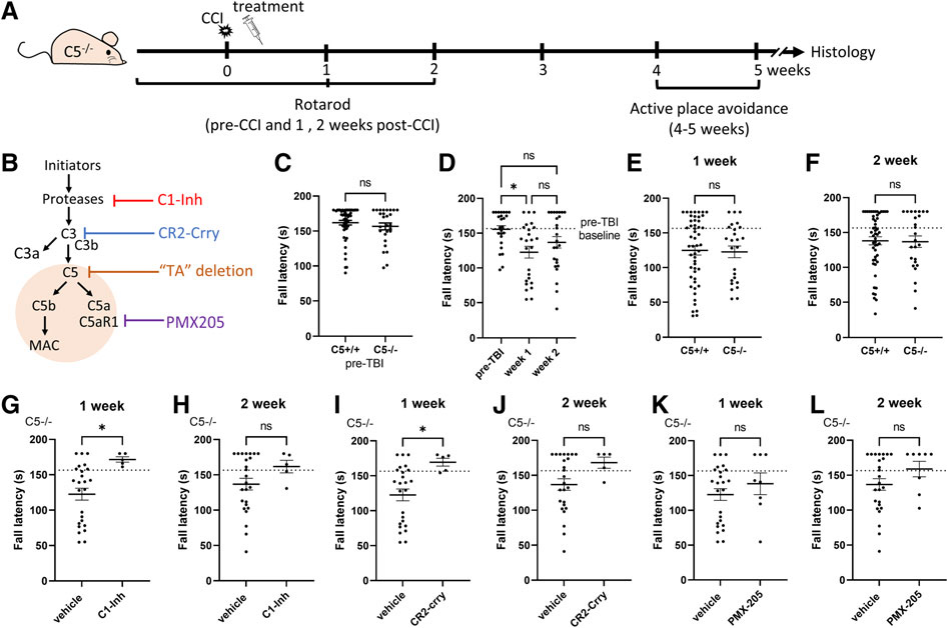
Effects of complement inhibitors on rotarod performance in C5^–/–^ mice, at 1 and 2 weeks after CCI injury. (**A**) Schematic representation of the timeline for the experimental procedures and drug administration. (**B**) Summary diagram of the complement pathways and site of action of the inhibitors and the “TA” deletion. (**C**) Rotarod performance was equivalent in each C5 genotype before CCI injury (C5^+/+^, *n* = 52; C5^–/–^, *n* = 25; Mann-Whitney U test, *p* = 0.41). (**D**) Fall latency of the rotarod test was significantly lower at 1 week after CCI compared to pre-treatment baseline in C5^–/–^ mice (*n* = 25; Kruskal-Wallis' test, *p* < 0.05; Dunn's multiple comparisons test, *p < 0.05). (**E,F**) Rotarod performance was equivalent in each C5 genotype at 1 and 2 weeks after CCI injury (C5^+/+^, *n* = 52; C5^–/–^, *n* = 25; Mann Whitney U test, *p* = 0.67 at 1 week post-CCI and *p* = 0.90 at 2 weeks post-CCI). (**G,H**) Compared with vehicle, C1-Inh treatment increased fall latency in C5^–/–^ mice at 1 week after CCI injury (C1-Inh, *n* = 5; vehicle, *n* = 25; Mann-Whitney U test, *p < 0.05 at 1 week post-CCI and *p* = 0.20 at 2 weeks post-CCI). (**I,J**) Compared with vehicle, CR2-Crry treatment increased fall latency in the rotarod test in C5^–/–^ mice at 1 week after CCI injury (CR2-Crry, *n* = 5; vehicle, *n* = 25; *p = 0.02 at 1 week post-CCI and *p* = 0.11 at 2 weeks post-CCI). (**K,L**) Compared with vehicle, PMX205 treatment did not significantly increase fall latency in the 2 weeks after CCI injury vehicle in C5^–/–^ mice (PMX205, *n* = 15; vehicle, *n* = 52; Mann-Whitney U test, *p* = 0.39 at 1 week post-CCI and *p* = 0.14 at 2 weeks post-CCI). C1-Inh, C1 esterase inhibitor; CCI, controlled cortical impact; CR2-Crry, complement receptor 2-complement receptor 1–related gene/protein.

First, we used enzyme-linked immunosorbent assay (ELISA) to compare the C5a level in brains of sham-injured and TBI mice (*n* = 8 in each group) at 4 h post-injury. Because the variation among animals was very high in the brain samples, we genotyped mice for the TA deletion reported in some outbred mouse strains (Fig. 1A). Then, we examined the treatment effects of C1-Inh, CR2-Crry, and PMX205 in C5^+/+^ and C5^–/–^ mice from three cohorts of animals (Figs. 2A and 3A). Motor function was assessed using the rotarod task before controlled cortical impact (CCI) injury and at 1 and 2 weeks after TBI. Spatial learning ability was examined using the active place avoidance (APA) task over a 5-day testing period at 5 weeks post-TBI. Size of the brain lesion was measured on sections stained with cresyl violet, obtained 16 weeks after TBI. Figures 2B and 3B are summary diagrams of the complement pathways and the site of action of the inhibitors used in C5^+/+^ and C5^–/–^ mice. Sample sizes of treatment groups and different assays are summarized in Supplementary Table S1.

### Animals

Outbred CD1 male mice (Envigo, Indianapolis, IN) were used in this study. Mice were housed individually under controlled laboratory conditions (12-h light/dark cycle, with lights on at 7:00 am, temperature 22°C ± 1°C, air humidity 50–60%) with *ad libitum* access to food and water. Experimental procedures were approved by the University of Queensland Animal Ethics Committee (Approval No.: CAI/300/17) and the Animal Care and Use Review Office of the U.S. Army Medical Research and Development Command. All experiments were conducted in accordance with the guidelines of the Australian National Health and Medical Research Council. Behavioral tests were performed by operators blinded to genotypes and treatments.

### Genotyping

DNA was extracted from ear-notch tissue samples using standard HotSHOT DNA extraction procedures. DNA sequencing and mutagenically separated polymerase chain reaction (MS-PCR) methods were used to identify the TA deletion. For sequencing, a 543-base-pair (bp) fragment from the C5 gene that encompassed the site of the potential TA deletion was amplified using PCR. Primer sequences were C5-F1: TAGGAATTAGTTAAATTGTCTAGGG and C5-R1: GATTCAGCTACTCGTAGTTAC. The PCR annealing temperature was 52°C. PCR fragments were sequenced for detection of the TA deletion as previously described.^17^ MS-PCR-amplified PCR fragments were detected by capillary electrophoresis.

### Controlled cortical impact injury model

Adult mice, 9–10 weeks of age, were subjected to a severe unilateral cortical contusion by computer-controlled impact delivered by a beveled steel tip 3 mm in diameter (TBI-0310; Precision Systems and Instrumentation, Fairfax, VA) as previously described.^18^ Briefly, mice were deeply anesthetized with a mixture of tiletamine/zolezepam (Zoletil^®^ 100, 50 mg/kg; Virbac, Carros, France) and xylazine (20 mg/kg; Troy Laboratories, Glendenning, NSW, Australia) administered, intraperitoneally (i.p.), before being placed in a stereotaxic frame (World Precision Instruments, LLC, Sarasota, FL). The skull was exposed by a midline incision, before a 4-mm craniotomy was made just lateral to the sagittal suture and centered between the bregma and lambda, to allow removal of the skullcap without damage to the dura. A CCI injury was subsequently delivered by compressing the cortex of the left hemisphere to a depth of 2.0 mm at a velocity of 5.0 m/s for a duration of 100 ms. The incision was sutured without cranioplasty. Sham controls underwent the craniotomy procedure, but did not receive the CCI injury.

### Drug treatments

A single dose of C1-Inh (15.0 IU; Berinert^®^; CSL Behring GmbH, Marburg, Germany) or 0.9% saline solution was administered, intravenously (i.v.), by the tail vein 1 h after the CCI injury; CR2-Crry (10 mg/kg) or phosphate-buffered saline (PBS) were administered, i.v., by the tail vein at 1 and 7 h post-CCI; and PMX205 (1 mg/kg, synthesized in-house^19^) or 5% glucose vehicle solution were administered by i.p. injection at twice-daily intervals, commencing 1 h post-TBI over a 10-day period. The i.v. injections were performed with mice under isoflurane anaesthesia, and the i.p. injections were administered with mice restrained. Doses and administration times were based on the available literature documenting the effectiveness of each complement inhibitor, or the known half-life of the agent.^10,14,20^ Control mice received the equivalent amount of vehicle at each dosing interval.

### Motor function

The rotarod apparatus (Ugo Basile, Comerio, Italy) was used to assess motor function. Mice underwent initial training on an accelerating rotarod (4–16 rpm over 60 sec) in three trials separated by 5-min intervals. Motor function was subsequently assessed commencing the day before CCI injury and at 1 and 2 weeks after TBI, in three trials on an accelerating rotarod (4–40 rpm over 180 sec) with a minimum 15-min intertrial interval. Average time to fall from the rotating cylinder in each trial was recorded as the fall latency.

### Active place avoidance task

The APA apparatus (Bio-Signal Group, Acton, MA) consisted of an elevated arena (diameter 77 cm) with a metal grid floor (bar spacing 5 mm and diameter 3 mm), surrounded by a transparent cylindrical boundary (height, 32 cm). A visual cue consisting of a large black and white symbol/shape (A3 in size) was located on each of the four walls that housed the APA apparatus. The arena rotated counterclockwise (1 rpm), and a 60-degree region of the grid, which remained constant in relation to the room coordinates, served as the shock zone. A mild electric foot shock (500 ms, 60 Hz, 0.6 mA at 1.5-sec intervals) was delivered when the mouse entered the shock zone.

Mice were handled for ∼30–60 sec daily in the week before testing. On the day before commencing testing, mice were placed in the arena without visual cues and shocks for 5 min, to allow habituation to the testing environment and APA apparatus. Mice subsequently underwent 10-min trials over 5 consecutive days. An overhead tracking camera recorded each trial. Trials were subsequently analyzed using Track Analysis software (Bio-Signal Group), to determine the number of shocks received and shock-zone entries.

### C5a enzyme-linked immunosorbent assay

Blood samples (∼0.5 mL) were collected by cardiac puncture before perfusion, transferred into ethylenediaminetetraacetic acid tubes, mixed immediately with 10 μL of complement serine protease inhibitor FUT175 (5 mg/mL; BD Biosciences, San Jose, CA) to prevent *ex vivo* activation,^21^ and stored on ice. Plasma was collected after centrifugation at 13,000*g* for 10 min at 4°C. Injured ipsilateral hemispheres were snap-frozen in liquid nitrogen and stored at −80°C, before being ground to fine powder using a mortar and pestle on dry ice. Powdered brain tissue was subsequently dissolved in 1 mL of NP-40 lysis buffer (Invitrogen, Carlsbad, CA) containing 1 mM of phenylmethylsulfonyl fluoride (reconstituted in in dimethyl sulfoxide; Sigma-Aldrich, St. Louis, MO), 92.6 μM of FUT175 (BD Biosciences), and 10 μL of protease inhibitor mixture (Sigma-Aldrich). Dissolved brain tissue samples were then vortexed for 1 min, left to stand on ice for 1 h, centrifuged at 13,000*g* for 30 min at 4°C, and the supernatant subsequently collected. Both plasma samples and brain tissue supernatants were stored at −80°C. Protein concentrations in brain tissue supernatants were determined using the bicinchoninic acid protein assay procedure (ThermoFisherScientific, Waltham, MA). C5a expression levels in plasma and brain tissue supernatants were determined by ELISA (R&D Systems mouse C5a DuoSet ELISA; R&D Systems, Minneapolis, MN), according to the manufacturer's instructions. C5a concentrations in brain tissue samples were adjusted to the protein concentration present in the sample and expressed as pg/μg protein.

### Tissue preparation for histological procedures

At 16 weeks post-TBI, mice were administered a lethal dose of sodium pentobarbital (600 mg/kg, i.p.) and perfused transcardially with 20 mL of PBS (pH, 7.4), followed by 30 mL of 4% paraformaldehyde dissolved in PBS. The brain was subsequently removed and post-fixed overnight in the same fixative solution, before being transferred to 15% and 30% sucrose in PBS for cryoprotection, frozen in FSC 22^®^ (Leica Microsystems, Wetzlar, Germany) frozen embedding medium on dry ice, and stored at −20°C until sectioning. Coronal sections (20 μm, between bregma −1.23 and −2.03 mm) were cut on a Leica cryostat, mounted directly to SuperFrostPlus^®^ slides, and subsequently stored at −20°C.

### Cresyl violet staining and assessment of lesion size

Series of every one in ten sections (20 μm thickness, 200 μm apart) were collected on one slide at the site of the injury. Slides were allowed to return to room temperature and briefly washed in deionized water (10–15 sec). They were then passed through 70% ethanol (3 min), 100% ethanol (2 × 3 min), and xylene (2 × 3 min) to defat the sections; before being rehydrated by passing back through 100% ethanol (2 × 1 min), 70% ethanol (1 min), and briefly rinsed in deionized water (10–15 sec). Sections were then transferred to 0.1% cresyl violet acetate in acetate buffer (5 mL of 1 M of sodium acetate in a final volume of 200 mL adjusted to pH: 3.85–3.90 with glacial acetic acid) for 5–10 min, before differentiation in 70% ethanol (30 sec), dehydration in 100% ethanol (2 × 1 min), and cleared in xylene (2 × 3 min). Sections were subsequently mounted with DPX^®^ neutral mounting medium. Lesion size was measured and averaged from three brain sections at anteroposterior levels −1.50, −1.70, and −1.90 mm from bregma using ImageJ (National Institutes of Health, Bethesda, MD).^22^ Percent area of tissue loss in the ipsilateral hemisphere was calculated as shown below.


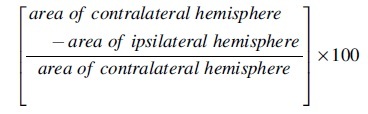


where ipsi- and contralateral respectively refer to the cerebral hemisphere ipsi- and contralateral to the site of injury.

### Statistical analysis

Statistical comparisons were performed using GraphPad Prism^®^ software (version 8.3; GraphPad Software Inc., La Jolla, CA). Normality of continuous data was assessed using the Shapiro-Wilk test, and homoscedasticity was assessed using Bartlett's test. Where the assumptions of normality and homoscedasticity were not violated, data were analyzed using unpaired Student's *t*-tests, one-way analysis of variance (ANOVA), or repeated-measures two-way ANOVA as appropriate. Non-parametric analyses were performed if these assumptions were violated as specified in the Results section. The criterion for statistical significance was *p* < 0.05. Data are presented as mean ± standard error of the mean values.

## Results

### C5 deficiency inhibited the activation of C5/C5a signaling after controlled cortical impact injury

We detected a TA deletion previously reported in C57BL/10Sn mice^17^ when we amplified and sequenced a 543-bp fragment from the C5 gene in CD1 mice (Fig. 1B). Genotypes were further confirmed with MS-PCR, which showed PCR fragments of two different sizes (Fig. 1C). In our study, of a total of 295 mice, 105 (35.6%) mice were C5 sufficient (C5^+/+^), 132 (44.8%) mice carried the TA deletion in one allele only (C5^+/–^), and 58 (19.6%) were C5 deficient with the TA deletion in both alleles (C5^–/–^).

We measured C5a, a protein fragment released from cleavage of C5 by C5 convertase, in the hemispheres ipsilateral to the site of injury, 4 h after CCI, using ELISA (Fig. 1D). Like other inbred C5-deficient mice, plasma C5a was undetectable in C5^–/–^ CD1 mice.^23^ In injured C5^+/+^ and C5^+/–^ mice, CCI increased C5a levels (∼8-fold) compared with sham surgery. After CCI, brain C5a, which requires secretion, was lower in C5^–/–^ mice than in C5^+/+^ (∼7-fold) and C5^+/–^ (∼6-fold) mice but still detectable. A possible explanation is that intracellular C5/C5a is present in brain tissue of C5^–/–^ mice and is released by brain injury. Brain C5a in CCI-injured C5^–/–^ mice (*n* = 3) was significantly higher than in sham-injured C5^+/+^ and C5^+/–^ mice (*n* = 8, *t*_(9)_ = 3.1, *p* = 0.013, unpaired Student's *t*-test), indicating that TBI induced the generation or release of C5a in injured C5^–/–^ mice.

### CR2-Crry, but not C1-Inh or PMX205, improved rotarod performance after controlled cortical impact injury

In this study, we examined the therapeutic effect of complement inhibitors on motor and cognitive functions in C5^+/+^ mice. The contribution of C5 to brain damage and the influences of C5 deficiency on the therapeutic impacts of complement inhibitors were studied using C5^–/–^ mice.

We investigated the effects of complement inhibitors on rotarod performance by comparing the effects of each inhibitor against the effects of vehicle treatment in C5^+/+^ mice. Because fall latency at 1 and 2 weeks after CCI was not significantly different between vehicle-treated groups (one-way ANOVA, *F*_(3, 48)_ = 0.74, *p* = 0.53 and *F*_(3, 47)_ = 2.00, *p* = 0.13), we combined data from all vehicle-treated mice. We examined rotarod performance in C5^+/+^ mice after CCI injury and found that fall latencies at 1 and 2 weeks after CCI were significantly lower than the pre-CCI baseline (Kruskal-Wallis' test, ****p < 0.0001; Dunn's multiple comparisons test, *p < 0.05, ****p < 0.0001; Fig. 2D). In the injured C5^+/+^ mice, CR2-Crry treatment improved rotarod performance to the pre-CCI baseline level at 1 week post-CCI, and fall latency of CR2-Crry-treated mice was significantly higher compared with vehicle-treated mice (Mann-Whitney U test, *p < 0.05; Fig. 2G,H). In the injured C5^+/+^ mice, fall latency over the 2-week testing period was not significantly affected by C1-Inh treatment (Mann-Whitney U test, *p* = 0.85 at 1 week post-CCI and *p* = 0.25 at 2 weeks post-CCI; Fig. 2E,F) and PMX205 treatment (Mann-Whitney U test, *p* = 0.74 at 1 week post-CCI and *p* = 0.24 at 2 weeks post-CCI; Fig. 2I,J).

Taken together, these findings indicate that inhibition of all complement pathways at the C3 level with CR2-Crry can accelerate the recovery of motor function. However, inhibition of the classic and lectin pathways with C1-Inh, and inhibition of C5aR1 signaling with PMX205, did not alleviate deficits in motor function post-TBI.

### C5 deficiency promoted the effects of C1-Inh and CR2-Crry on the recovery of motor function

We compared rotarod performance between C5^+/+^ and C5^–/–^ mice before CCI injury and observed no significant difference (Mann-Whitney U test, *p* = 0.41; Fig. 3C), indicating that these genotypes had no effect on motor function in CD1 mice, as is the case for other inbred C5-deficient mouse strains.^24^ Because fall latency at 1 and 2 weeks after CCI was not significantly different between vehicle-treated groups (one-way ANOVA, *F*_(3, 20)_ = 0.6073, *p* = 0.62 and *F*_(3, 21)_ = 2.55, *p* = 0.083), we combined data from all vehicle-treated mice. In the injured C5^–/–^ mice, fall latencies at 1 week, but not at 2 weeks, after CCI were significantly lower than the pre-CCI baseline (Kruskal-Wallis' test, *p < 0.05; Dunn's multiple comparisons test, *p < 0.05; Fig. 3D). We examined rotarod performance in C5^+/+^ and C5^–/–^ mice after CCI injury by comparing fall latency at 1 and 2 weeks post-CCI and observed no significant main effect on fall latency for genotype (Mann-Whitney U test, *p* = 0.67 at 1 week post-CCI and *p* = 0.90 at 2 weeks post-CCI; Fig. 3E,F), suggesting that C5 deficiency does not alter the recovery of motor function after CCI.

In the injured C5^–/–^ mice, we also observed a significant increase in fall latency at 1 week after CCI for CR2-Crry-treated mice compared with vehicle-treated mice (Mann-Whitney U test, *p = 0.02 at 1 week post-CCI and *p* = 0.11 at 2 weeks post-CCI; Fig. 3I,J). Though C1-Inh failed to improve the recovery of motor function after TBI in C5^+/+^ mice, in the injured C5^–/–^ mice treated with C1-Inh, fall latency of C1-Inh-treated mice was significantly higher compared with vehicle-treated mice at 1 week after CCI (Mann-Whitney U test, *p < 0.05 at 1 week post-CCI and *p* = 0.20 at 2 weeks post-CCI; Fig. 3G,H), suggesting that C5 deficiency enhanced the treatment effect of C1-Inh. Similar to injured C5^+/+^ mice, there was no significant effect on fall latency in the rotarod test after treatment with PMX205 in CCI-injured C5^–/–^ mice (Mann-Whitney U test, *p* = 0.39 at 1 week post-CCI and *p* = 0.14 at 2 weeks post-CCI; Fig. 3K,L).

### CR2-Crry and PMX205, but not C1-Inh, improved performance in the active place avoidance task after controlled cortical impact injury

**FIG. 4.**
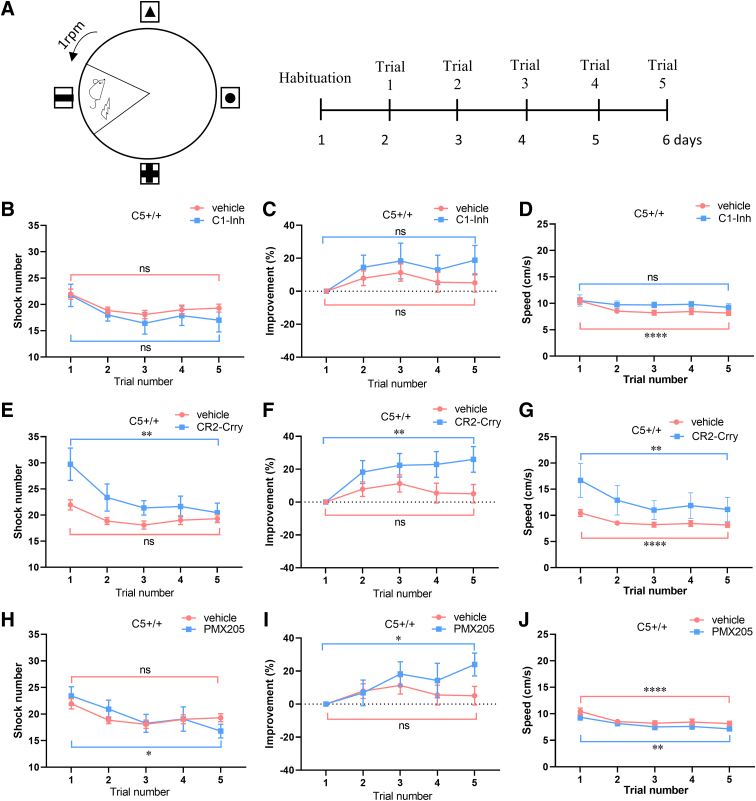
Effects of complement inhibitors on performance in the APA task over 5 days, in C5^+/+^ mice after CCI injury. (**A**) Setup in the APA test. (**B,C**) In the injured C5^+/+^ mice treated with vehicle or C1-Inh, there were no significant differences in shock number (C1-Inh, *n* = 7; vehicle, *n* = 52; vehicle: Friedman's test, **p < 0.01; Dunn's multiple comparisons test, *p* = 0.33; C1-Inh: Friedman's test, *p* = 0.37; Dunn's multiple comparisons test, *p* = 0.36) and percent improvement (vehicle: Friedman's test, **p < 0.01; Dunn's multiple comparisons test, *p* = 0.20 and C1-Ihn: Friedman's test, *p* = 0.41; Dunn's multiple comparisons test, *p* = 0.43) between day 5 and day 1. (**D**) Average velocity was slightly, but significantly, lower on day 5 than day 1 in injured C5^+/+^ mice treated with vehicle (Friedman's test, ****p < 0.0001; Dunn's multiple comparisons test, ****p < 0.0001), but not in injured C5^+/+^ mice treated with C1-Inh (Friedman's test, *p* = 0.44; Dunn's multiple comparisons test, *p* = 0.25). (**E,F**) In the injured C5^+/+^ mice treated with CR2-Crry, shock number was reduced (CR2-Crry, *n* = 11; Friedman's test, *p < 0.05; Dunn's multiple comparisons test, **p < 0.01;) and percent improvement was increased (Friedman's test, *p < 0.05; Dunn's multiple comparisons test, **p < 0.01) on day 5 compared with day 1. (**G**) In the injured C5^+/+^ mice treated with CR2-Crry, there was a significant increase in average velocity on day 5 compared with day 1 (Friedman's test, **p < 0.01; Dunn's multiple comparisons test, **p < 0.01). (**H,I**) PMX205 treatment also reduced shock number in injured C5^+/+^ mice (PMX205, *n* = 15; Friedman's test, *p < 0.05; Dunn's multiple comparisons test, *p < 0.05) and increased percent improvement (Friedman's test, *p < 0.05; Dunn's multiple comparisons test, *p < 0.05). (**J**) In the injured C5^+/+^ mice treated with PMX205, there was a significant increase in average velocity on day 5 compared with day 1 (Friedman's test, **p < 0.01; Dunn's multiple comparisons test, **p < 0.01). APA, active place avoidance; C1-Inh, C1 esterase inhibitor; CCI, controlled cortical impact; CR2-Crry, complement receptor 2-complement receptor 1–related gene/protein.

APA is a challenging spatial learning task that relies on hippocampal function. In a 5-day APA testing paradigm, TBI mice made significantly more mistakes than sham-operated mice and failed to show an improvement in performance over time.^25^ We assessed spatial learning ability using the APA task over a 5-day testing period at 5 weeks after CCI injury (Fig. 4A). Improvement during the APA task was analyzed by calculating the reduction in shock number compared to the number on the first trial in each mouse and expressing this value as a percentage. Average velocity was used to examine the movement during APA testing. Among vehicle-treated groups, there were no significant main effects on shock number (repeated-measures two-way ANOVA, *F*_(3, 73)_ = 2.50, *p* = 0.066), percent improvement (repeated-measures two-way ANOVA, *F*_(3, 73)_ = 2.36, *p* = 0.23), and velocity during the 5-day testing period (repeated-measures two-way ANOVA, *F*_(3, 69)_ = 0.67, *p* = 0.57). Hence, we combined data from all vehicle-treated mice and compared them with the data sets for each complement inhibitor treatment.

To test the improvement in performance over time in injured C5^+/+^ mice treated with vehicle or complement inhibitors, we compared shock numbers, percent improvement, and average velocity on day 5 with day 1. In injured C5^+/+^ mice treated with vehicle, there were no significant differences in shock number (Friedman's test, **p < 0.01; Dunn's multiple comparisons test, *p* = 0.33; Fig. 4B,E,H) and percent improvement (Friedman's test, **p < 0.01; Dunn's multiple comparisons test, *p* = 0.20; Fig. 4C,F,I) between day 5 and day 1. In injured C5^+/+^ mice treated with vehicle, average velocity was slightly, but significantly, lower on day 5 than day 1 (Friedman's test, ****p < 0.0001; Dunn's multiple comparisons test, ****p < 0.0001; Fig. 4D,G,J).

C1-Inh treatment did not significantly affect shock number (Friedman's test, *p* = 0.37; Dunn's multiple comparisons test, *p* = 0.36; Fig. 4B) and percent improvement (Friedman's test, *p* = 0.41; Dunn's multiple comparisons test, *p* = 0.43; Fig. 4C). In C1-Inh treatment, there was also no difference in average velocity on day 5 compared with day 1 (Friedman's test, *p* = 0.44, Dunn's multiple comparisons test, *p* = 0.25; Fig. 4D). In injured C5^+/+^ mice treated with CR2-Crry, shock number was reduced (Friedman's test, *p < 0.05, Dunn's multiple comparisons test, **p < 0.01; Fig. 4E) and percent improvement was increased (Friedman's test, *p < 0.05, Dunn's multiple comparisons test, **p < 0.01; Fig. 4F) on day 5 compared with day 1. PMX205 treatment also reduced shock number (Friedman's test, *p < 0.05; Dunn's multiple comparisons test, *p < 0.05; Fig. 4H) and increased percent improvement (Friedman's test, *p < 0.05; Dunn's multiple comparisons test, *p < 0.05; Fig. 4I). In the injured C5^+/+^ mice treated with CR2-Crry and PMX205, there was a significant increase in average velocity on day 5 compared with day 1 (Friedman's test, **p < 0.01; Dunn's multiple comparisons test, **p < 0.01; Fig. 4G and Friedman's test, **p < 0.01; Dunn's multiple comparisons test, **p < 0.01; Fig. 4J).

Taken together, these findings support a role for complement activation at the level of C3 or C5aR1 in the loss of cognitive function after CCI injury.

### C5 deficiency improved performance in the active place avoidance task, but did not further enhance the treatment effects of C1-Inh, CR2-Crry, and PMX205

**FIG. 5.**
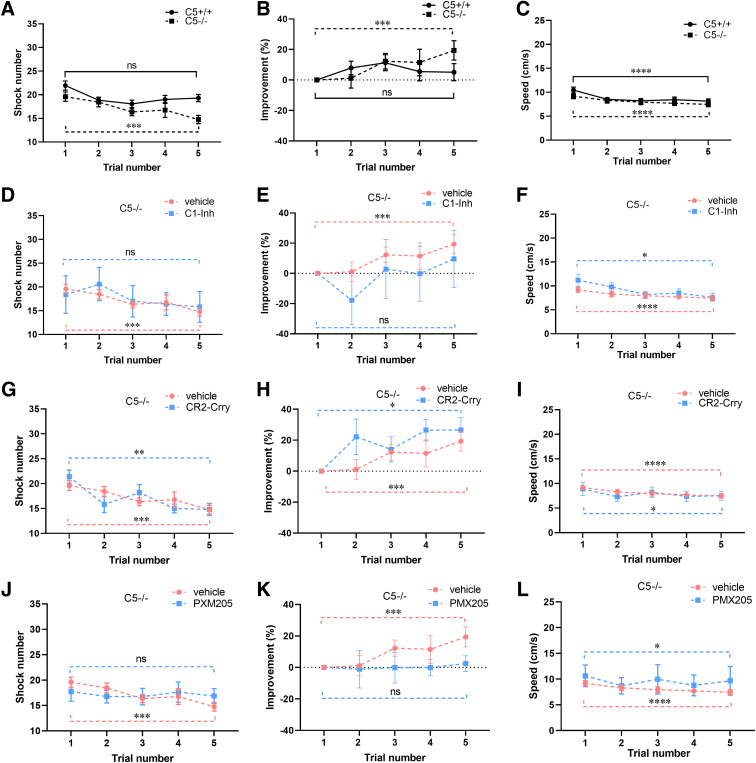
Effects of complement inhibitors on performance in the APA task over 5 days, in C5^–/–^ mice after CCI injury. (**A**) Shock-zone entries were significantly lower on day 5 compared with day 1 in C5^–/–^ mice (C5^–/–^, *n* = 25; Friedman's test, **p < 0.01; Dunn's multiple comparisons test, ***p < 0.001), but not in C5^+/+^ mice (C5^+/+^, *n* = 52; Friedman's test, **p < 0.01; Dunn's multiple comparisons test, *p* = 0.33). (**B**) Percent improvement was higher on day 5 compared with day 1 in C5^–/–^ mice (Friedman's test, **p < 0.01; Dunn's multiple comparisons test, ***p < 0.01), but not in C5^+/+^ mice (Friedman's test, **p < 0.01; Dunn's multiple comparisons test, *p* = 0.20). (**C**) Average velocity was reduced on day 5 in both C5^+/+^ and C5^–/–^ mice after CCI (C5^+/+^, *n* = 52; Friedman's test, ****p < 0.0001; Dunn's multiple comparisons test, ****p < 0.0001; C5^–/–^, *n* = 25; Friedman's test, ***p < 0.001; Dunn's multiple comparisons test, ****p < 0.0001). (**D–F**) In the injured C5^–/–^ mice treated with C1-Inh, shock number (C1-Inh, *n* = 5; Friedman's test, *p* = 0.12; Dunn's multiple comparisons test, *p* = 0.65) and percent improvement (Friedman's test, *p* = 0.12; Dunn's multiple comparisons test, *p* = 0.65) were not significantly changed, but average velocity was reduced (Friedman's test, *p < 0.05; Dunn's multiple comparisons test, *p < 0.05) on day 5 compared with day 1. (**G–I**) In the injured C5^–/–^ mice treated with CR2-Crry, shock number was reduced (CR2-Crry, *n* = 5; Friedman's test, **p < 0.01; Dunn's multiple comparisons test, **p < 0.01), percent improvement was increased (Friedman's test, *p < 0.05; Dunn's multiple comparisons test, *p < 0.05), and average velocity was reduced (Friedman's test, *p < 0.05; Dunn's multiple comparisons test, *p < 0.05) on day 5 compared with day 1. (**J–L**) In the injured C5^–/–^ mice treated with PMX205, shock number (PMX205, *n* = 8; Friedman's test, *p* = 0.12; Dunn's multiple comparisons test, *p* = 0.99) and percent improvement (Friedman's test, *p* = 0.49; Dunn's multiple comparisons test, *p* = 0.99) were not significantly changed, but average velocity was reduced (Friedman's test, *p < 0.05; Dunn's multiple comparisons test, *p < 0.05), on day 5 compared with day 1. APA, active place avoidance; C1-Inh, C1 esterase inhibitor; CCI, controlled cortical impact; CR2-Crry, complement receptor 2-complement receptor 1–related gene/protein.

To test for the effects of genotype, we compared shock numbers, percent improvement, and average velocity of the fifth trial with the first trial in vehicle-treated C5^+/+^ and C5^–/–^ mice. Shock number was significantly lower and percent improvement was higher on day 5 compared with day 1 in injured C5^–/–^ mice treated with vehicle (Friedman's test, **p < 0.01; Dunn's multiple comparisons test, ***p < 0.001; Fig. 5A and Friedman's test, **p < 0.01; Dunn's multiple comparisons test, ***p < 0.01; Fig. 5B). In the injured C5^–/–^ mice treated with vehicle, average velocity was also slightly, but significantly, lower on day 5 than in day 1 (Friedman's test, ***p < 0.001; Dunn's multiple comparisons test, ****p < 0.0001; Fig. 5C). These findings indicate that C5 deficiency attenuates loss of cognitive function after CCI injury.

We also examined the effects of C1-Inh, CR2-Crry, and PMX205 on performance in the APA task in C5^–/–^ mice after CCI injury and observed reduction in shock number and increase in percent improvement on day 5 compared with day 1 in CR2-Crry treatment (Friedman's test, **p < 0.01; Dunn's multiple comparisons test, **p < 0.01; Fig. 5G and Friedman's test, *p < 0.05; Dunn's multiple comparisons test, *p < 0.05; Fig. 5H). Given that C5 deficiency attenuated the loss of cognitive function in C5^–/–^ mice after CCI injury (Fig. 5A,B) and group sizes for C5^–/–^ mice were relatively small, no further improvement in the APA task was observed when these treatments were compared with their respective vehicle controls. C1-Inh and PMX205 treatment did not reduce shock number (Friedman's test, *p* = 0.12; Dunn's multiple comparisons test, *p* = 0.65; Fig. 5D and Friedman's test, *p* = 0.12; Dunn's multiple comparisons test, *p* = 0.99; Fig. 5J) or increase percent improvement (Friedman's test, *p* = 0.12; Dunn's multiple comparisons test, *p* = 0.65; Fig. 5E and Friedman's test, *p* = 0.49; Dunn's multiple comparisons test, *p* = 0.99; Fig. 5K) on day 5 in injured C5^–/–^ mice.

Like vehicle treatment, average velocity was also reduced on day 5 compared to day 1 in injured C5^–/–^ mice treated with these treatments (Friedman's test, *p < 0.05; Dunn's multiple comparisons test, *p < 0.05; Fig. 5F; Friedman's test, **p < 0.01; Dunn's multiple comparisons test, *p < 0.05; Fig. 5I; and Friedman's test, *p < 0.05; Dunn's multiple comparisons test, *p < 0.05; Fig. 5L).

### PMX205, but not C1-Inh and CR2-Crry, reduced lesion size after controlled cortical impact injury

**FIG. 6.**
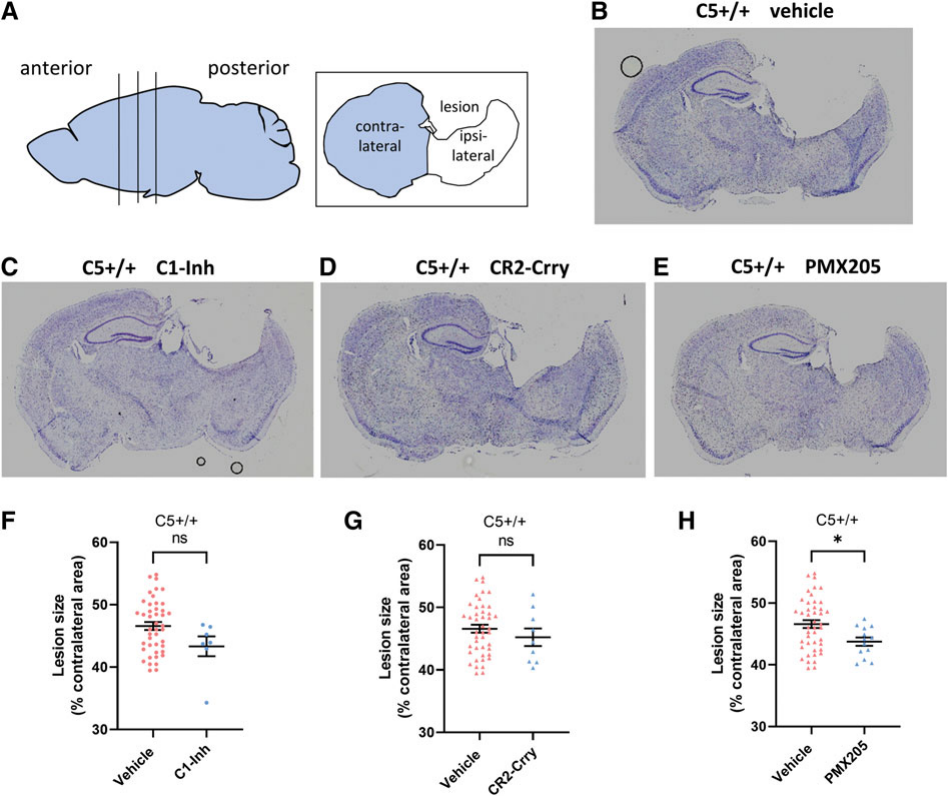
Effects of complement inhibitors on lesion size in C5^+/+^ mice. (**A**) Data analysis utilized brain sections at three anteroposterior levels (−1.50, -1.70, and −1.90 mm from bregma). (**B–E**) Representative images of brain sections from different treatment groups. (**F**) In C5^+/+^ mice, lesion size was not significantly different after treatment with C1-Inh compared with vehicle (C1-Inh, *n* = 7; vehicle, *n* = 44). (**G**) In C5^+/+^ mice, lesion size was not significantly different after treatment with CR2-Crry compared with vehicle (CR2-Crry, *n* = 9; vehicle, *n* = 44). (**H**) In C5^+/+^ mice, lesion size was significantly lower after treatment with PMX205 compared with vehicle (PMX205, *n* = 13; vehicle, *n* = 44; unpaired Student's *t*-test, *p < 0.05). C1-Inh, C1 esterase inhibitor; CR2-Crry, complement receptor 2-complement receptor 1–related gene/protein.

We measured lesion size using percentage area of the hemisphere ipsilateral to the site of impact that was injured on brain sections stained with cresyl violet (Fig. 6A–E) at 16 weeks after CCI injury. There was no significant difference in lesion size between vehicle-treated groups (one-way ANOVA, *F*_(3,58)_ = 2.57, *p* = 0.06); hence, the effect of each complement inhibitor on lesion size was compared against pooled data for their vehicle treatments. In the injured C5^+/+^ mice, lesion size was significantly reduced after treatment with PMX205 (*t*_(55)_ = 2.28, *p* = 0.027, unpaired Student's *t*-test; Fig. 6H), but not after treatment with C1-Inh (*t*_(49)_ = 1.88, *p* = 0.07, unpaired Student's *t*-test; Fig. 6F) and CR2-Crry (*t*_(51)_ = 0.87, *p* = 0.39, unpaired Student's *t*-test; Fig. 6G).

### C5 deficiency enhanced the effects of C1-Inh and CR2-Crry on brain damage

**FIG. 7.**
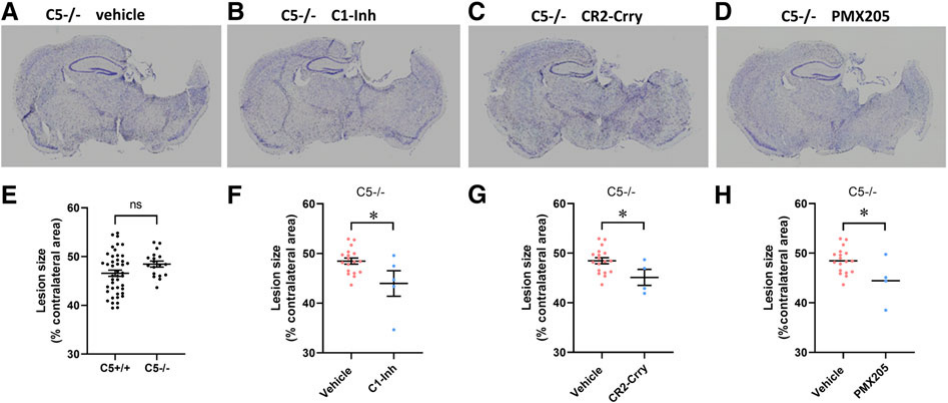
Effects of complement inhibitors on lesion size in C5^–/–^ mice. (**A–D**) Representative images of brain sections from different treatment groups. (**E**) Lesion size was not significantly different between vehicle-treated C5^+/+^ and C5^–/–^ mice (C5^+/+^, *n* = 44; C5^–/–^, *n* = 18; unpaired Student's *t*-test). (**F**) In C5^–/–^ mice, lesion size was significantly lower after treatment with C1-Inh compared with vehicle (C1-Inh, *n* = 5; vehicle, *n* = 18; unpaired Student's *t*-test, *p < 0.05). (**G**) In C5^–/–^ mice, lesion size was significantly lower after treatment with CR2-Crry compared with vehicle (CR2-Crry, *n* = 4; vehicle, *n* = 18; unpaired Student's *t*-test, *p < 0.05). (**H**) Lesion size was significantly lower after treatment with PMX205 than after vehicle treatment (PMX205, *n* = 4; vehicle, *n* = 18; unpaired Student's *t*-test, *p < 0.05) in C5^–/–^ mice. C1-Inh, C1 esterase inhibitor; CR2-Crry, complement receptor 2-complement receptor 1–related gene/protein.

To assess the effect of C5 genotype on lesion size we compared lesion size between injured C5^+/+^ and C5^–/–^ vehicle-treated mice and found no significant difference (*t*_(60)_ = 1.74, *p* = 0.087, unpaired Student's *t*-test; Fig. 7E). In the injured C5^–/–^ mice, lesion size was significantly reduced after treatment with C1-Inh, CR2-Crry, and PMX205 (unpaired Student's *t*-test; *t*_(21)_ = 2.62, *p* = 0.016, Fig. 7F; *t*_(20)_ = 2.27, *p* = 0.034, Fig. 7G; *t*_(20)_ = 2.45, *p* = 0.023; Fig. 7H), suggesting that C1-Inh and CR2-Crry had a neuroprotective effect after TBI in C5^–/–^ mice.

## Discussion

**FIG. 8.**
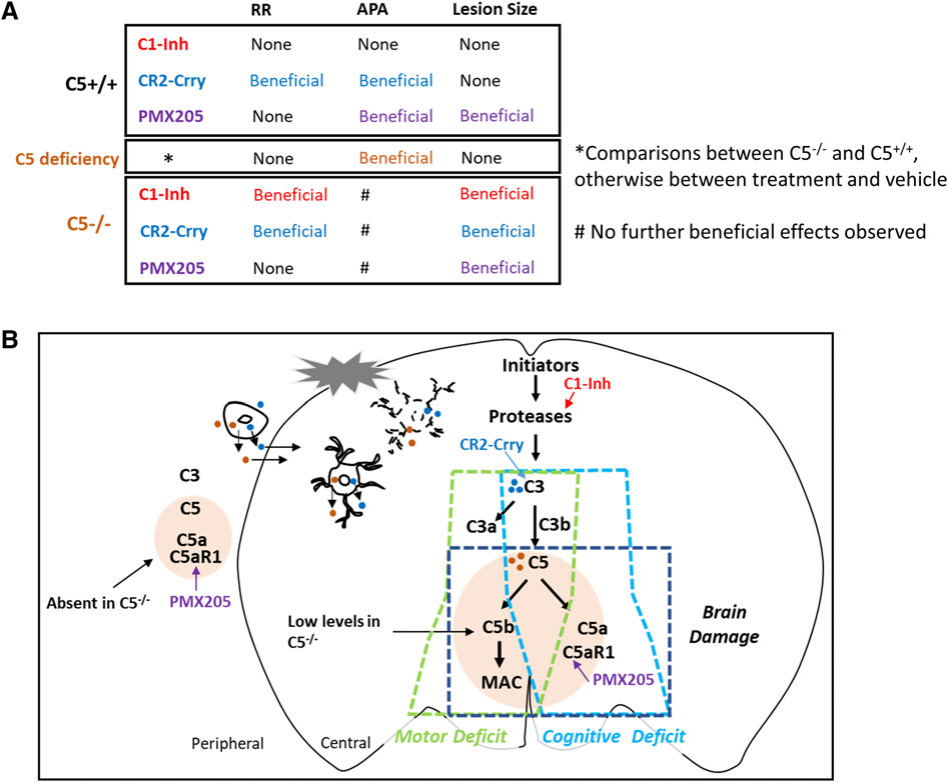
Effects of complement inhibitors in CCI injury. (**A**) Summary of the treatment effects of complement inhibitors after CCI injury in the present study. (**B**) Role of the complement cascade in functional deficits and brain damage after CCI injury. APA, active place avoidance; C1-Inh, C1 esterase inhibitor; CCI, controlled cortical impact; RR, rotarod.

In the present study, we examined the effects of C1-Inh, CR2-Crry, and PMX205 on motor and cognitive function, and brain lesion size after TBI in C5^+/+^ and C5^–/–^ mice. As summarized in Figure 8A, CR2-Crry improved rotarod and APA performances, and PMX205 improved APA performance and reduced brain lesion size in C5^+/+^ mice. C5 deficiency improved performance in the APA task after CCI injury. C5 deficiency enhanced the effect of C1-Inh on rotarod performance. C1-Inh and CR2-Crry also reduced brain lesion size in C5^–/–^ mice.

### C3 degradation products C3a/C3b and membrane attack complex signaling are the main drivers of motor deficits

In C5^+/+^ mice, we showed that CR2-Crry treatment improved motor function after TBI, whereas PMX-205 and C5 deficiency had no significant effects, supporting the critical role of C3a/C3b in motor deficits post-TBI (Fig. 8B). CR2-Crry specifically targets sites of C3d deposition in the brain and transiently inhibits complement activation in the acute phase, thereby limiting systemic adverse effects of complement inhibition and the interruption of complement activation during recovery.^26–28^ The therapeutic efficacy of CR2-Crry has been assessed using a battery of motor function tasks post-TBI in the mouse CCI model.^10^ Treatment with CR2-Crry substantially improved motor function and suppressed microgliosis and astrogliosis post-TBI.^10,11^ In this study, CR2-Crry treatment improved recovery in the rotarod test of motor function, further supporting the critical role of C3 in motor deficits post-TBI.

In previous research, administration of C1-Inh 1 h after CCI injury in mice attenuated motor deficits, assessed using a composite neuroscore that included lateral pulsion, forelimb function, hindlimb function, and the angle board test.^29^ In a rat weight-drop model, treatment with C1-Inh before TBI induction reduced brain edema 2 days after injury.^30^ These studies have attributed the beneficial effects of C1-Inh to inhibition of the complement system, even though brain C3a levels were reduced by only 15% after treatment with C1-Inh.^30^ However, as assessed in the rotarod test, we found that C1-Inh administered 1 h after CCI injury did not improve grip strength, balance, and motor coordination. This discrepancy may reflect the different methods used to assess motor function.

Although C5 deficiency and PMX205 treatment by themselves did not affect recovery in the rotarod test, C5 deficiency further enhanced the effect of C1-Inh on motor function. Our findings support a downstream role for MAC signaling. Our ELISA data demonstrated the presence of C5a in brain tissue in C5^–/–^ mice, perhaps reflecting release from damaged brain cells. It appears that even reduced amounts of C5/C5a released in the brain after TBI contribute to motor deficits in C5^–/–^ mice. Whereas blockade of the C5a receptor with PMX205 did not have a significant effect, we postulate that further reductions in MAC signaling from targeting upstream of the complement system with C1-Inh was sufficient to improve motor function in C5^–/–^ mice.

### The C3/C5/C5a receptor axis plays a detrimental role in cognitive function after traumatic brain injury

Consistent with previous studies in mice where treatment with C1-Inh 1 h after CCI injury did not improve cognitive function assessed using the Morris water maze,^29^ we found that treatment with C1-Inh did not improve performance in the APA task. We showed that CR2-Crry improved performance in the APA task after CCI injury in mice, supporting the key role of C3 in cognitive deficits after TBI. Treatment with CR2-Crry has also been reported to substantially improve cognitive function assessed using the Barnes maze.^10^

Complement activation upstream of C3 promotes chronic TBI neuropathology, but the contribution of downstream molecules is still unclear. The use of C5-deficient mouse strains has led to significant progress in understanding normal and pathological functions of the C5 gene.^24,31^ Previous work in a TBI cryoinjury model has shown that neutrophil extravasation was significantly reduced in C5^–/–^ mice, but the study did not address whether C5 deficiency affected functional outcomes.^16^ By comparing C5^+/+^ and C5^–/–^ CD1 mice after CCI injury, we found that C5 deficiency significantly improved performance in the APA task.

Inhibition of the terminal MAC using CR2-CD59 post-CCI injury in mice was found to reduce acute deficits, but not prevent chronic deficits, indicating that opsonins and anaphylatoxins contributed to chronic neuroinflammation in a MAC-independent manner.^10^ In a previous study, C5aR1 mRNA expression was upregulated in cerebellar Purkinje cells in rats after diffuse axonal injury,^32^ whereas a C5aR1 antagonist peptide reduced neutrophil extravasation in a cryoinjury mouse model, suggesting that inhibition of the C5a receptors may ameliorate TBI.^16^ We examined the specific role of the C5aR1 in cognitive function and found that inhibition of C5aR1 using PMX205 improved performance in the APA task. Treatment with CR2-Crry and PMX205 and C5 deficiency improved performance in the APA task, indicating that activation of the C3/C5/C5aR1 axis all play detrimental roles in cognitive function post-TBI (Fig. 8B).

### C5/C5a/C5aR1 signaling contributes to brain damage

We found that brain lesion size did not differ significantly between C5^+/+^ and C5^–/–^ mice post-CCI injury whereas treatment with PMX205 reduced brain lesion in both C5^–/–^ and C5^+/+^ mice. These findings suggest a key role for C5aR1 in brain damage. That PMX205 also reduced brain lesion in C5^–/–^ mice is in keeping with the ELISA data demonstrating the presence of low levels of C5a in brain tissue in these animals. Even these small amounts of brain C5/C5a and a low level of C5aR1 activation could be sufficient for inducing brain damage, potentially also explaining the protective effects of C1-Inh and CR2-Crry in C5^–/–^ mice. In C5^+/+^ mice, it is likely that targeting upstream parts of the complement cascade, using C1-Inh and CR2-Crry, do not inhibit downstream C5aR1 signaling fully, whereas in C5^–/–^ mice, the combination of C5 deficiency with C1-Inh or CR2-Crry provides more effective inhibition of C5aR1 signaling resulting in brain-protective effects. In previous studies, MAC inhibition significantly reduced lesion volume at 30 days after TBI, but the effect is less robust compared with the inhibition of C3.^10^ These findings support a critical role for downstream elements of the complement system in brain damage post-TBI.

Although treatment with CR2-Crry5 and PMX205 in C5^+/+^ mice, as well as C5 deficiency, improved performance in the APA task, it appears from the histological images (Figs. 6 and 7) that the hippocampus is still severely damaged after these treatments. Spatial cognition involves a network of structures, such as the medial pre-frontal cortex, in addition to the ventral hippocampus.^33^ We focused on the hippocampus in this study and did not observe the protective effect on the hippocampus, suggesting that improved performance after CR2-Crry and PMX205 treatment are related to protective effects elsewhere in the network.

### Limitations

A limitation of our study is that we did not validate the effects of C5 deficiency by using C5 inhibitors, such as C5 targeting monoclonal antibodies. It is possible that deep anesthesia during surgery added a component of hypoxic brain injury, and during drug administration, depending on the treatment, some animals had an additional inhalational anesthetic agent or were restrained, which may alter inflammation/immune function. However, we consider the potential degree of added variability to be mild and unlikely to negate our findings.

### Future direction

Our study demonstrated that C3 inhibition alleviates both motor and cognitive deficits after TBI, suggesting CR2-Crry as a potent therapeutic strategy in TBI. Our findings also suggest that even a low level of C5 released in damaged brain tissue is sufficient to contribute to motor deficits and brain damage. Upstream targeting of the complement cascade by CR2-Crry may not achieve adequate inhibition of terminal components; C5 inhibition/deficiency enhanced the treatment effects of CR2-Crry on brain damage. The findings suggest that combination of C3 and C5 inhibitors as an attractive therapeutic strategy for TBI. A previous study demonstrated that alternative completement pathway inhibitor CR2-fH provided similar beneficial effects to CR2-Crry in the CCI model.^10^ A few humanized C5 antibodies have been approved for clinical use or are advanced in their clinical development. For instance, eculizumab and ravulizumab (ALXN210) are FDA approved for treating paroxysmal nocturnal hemoglobinuria.^34,35^ BB5.1 is a mouse analogue to eculizumab and ravulizumab, and future studies should examine the treatment effects of the combination therapy of BB5.1 and C3 inhibitors on TBI outcomes.

## Conclusion

We evaluated the effectiveness of treatment with three complement inhibitors targeting different levels of the complement system in both C5^+/+^ and C5^–/–^ CD1 mice post-CCI injury. Our findings suggest a combination treatment using a C5 inhibitor together with CR2-Crry as a potential novel therapeutic strategy for TBI. In addition, this study demonstrates that genetic variability in CD1 mice is a fact that can affect their response to injury.

## Supplementary Material

Supplemental data

## Transparency, Rigor, and Reproducibility Summary

The study and analysis plan were not formally registered. However, a rigorous specification of the hypotheses, design, and analysis plans were reviewed by the funding agency and detailed in the Statement of Work submitted to the agency. In addition, the research methodology was reviewed by the institutional animal research ethics committee, which also takes into account the scientific rigor of the proposed work. All studies were designed to detect significance at *p* < 0.05 with a power of ≥0.8 (G*Power 3.1.9.2). The actual sample sizes of treatment groups and different assays are summarized in Supplementary Table S1. Data generated or analyzed during the study are included in this published article. The data sets used and/or analyzed during the current study are available from the corresponding author on reasonable request. A limited number of histological samples from each experimental group will also be made available for future analyses on request. This article will be published under a Creative Commons Open Access license, and upon publication will be available at https://www.liebertpub.com/loi/neu.

## Abbreviations Used

ANOVA: analysis of variance
APA: active place avoidance
bp: base pair
C1-Inh: C1 esterase inhibitor
C3: complement component 3
C5: complement component 5
C5a: complement C5a
C5aR1: complement C5a receptor 1
CR2-Crry: complement receptor 2-complement receptor 1–related gene/protein
CCI: controlled cortical impact
ELISA: enzyme-linked immunosorbent assay
FDA: U.S. Food and Drug Administration
i.p.: intraperitoneal
i.v.: intravenous
MAC: membrane attack complex
mRNA: messenger RNA
MS-PCR: mutagenically separated polymerase chain reaction
PBS: phosphate-buffered saline
TBI: traumatic brain injury
