# Supplementary figures and images for: Complement Component 5 (C5) Deficiency Improves Cognitive Outcome After Traumatic Brain Injury and Enhances Treatment Effects of Complement Inhibitors C1-Inh and CR2-Crry in a Mouse Model
Source: Neurotrauma Rep. 2023 Oct 11;4(1):663–81. doi: 10.1089/neur.2023.0024 (PMC10615070; doi:10.1089/neur.2023.0024)

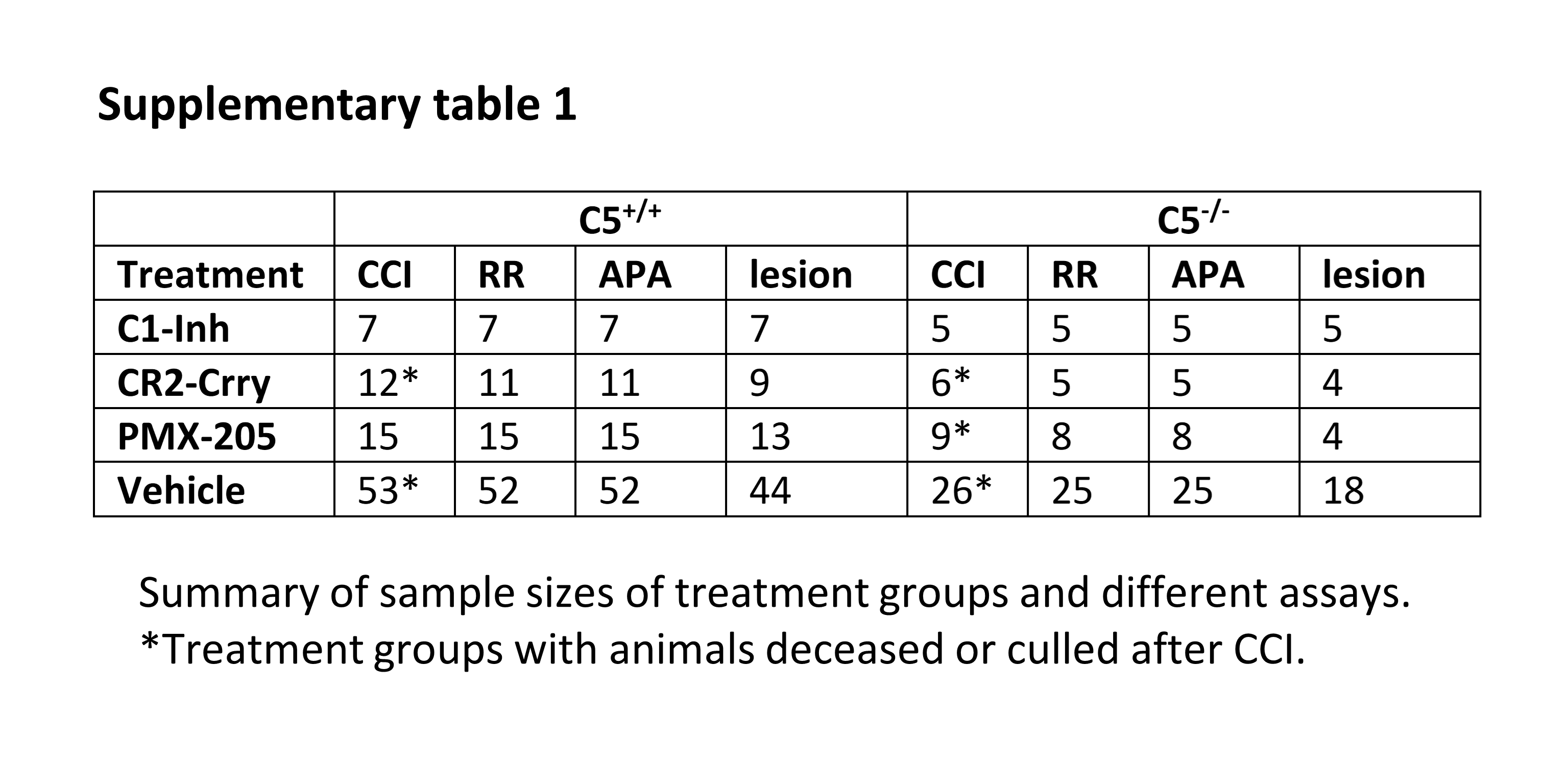

Supplement: Supplemental data [file Suppl_TableS1.TIF]
